# Outcome-Affecting Parameters of Hip Arthroscopy for Femoroacetabular Impingement with Concomitant Cartilage Damage—Data Analysis from the German Cartilage Registry

**DOI:** 10.3390/jcm11061532

**Published:** 2022-03-10

**Authors:** Sebastian Serong, Stefan Fickert, Philipp Niemeyer, Ingo J. Banke, Jens Goronzy, Christian Sobau, Wolfgang Zinser, Stefan Landgraeber

**Affiliations:** 1 Department of Orthopaedics & Orthopaedic Surgery, Saarland University Medical Center, 66424 Homburg, Germany; stefan.landgraeber@uks.eu; 2 Sporthopaedicum Straubing, 94315 Straubing, Germany; fickert@sporthopaedicum.de; 3 Department of Orthopedic Surgery and Traumatology, Mannheim University Hospital, 68167 Mannheim, Germany; 4 OCM Clinic Munich, 81369 Munich, Germany; phniemeyer@gmail.com; 5 Department of Orthopedics and Trauma Surgery, Freiburg University Hospital, 79106 Freiburg im Breisgau, Germany; 6 Clinic of Orthopedics and Sports Orthopedics, Klinikum Rechts der Isar, Technical University of Munich, 81675 Munich, Germany; ingo.banke@mri.tum.de; 7 Department of Orthopedics, Trauma and Plastic Surgery, University Hospital Carl Gustav Carus, 01307 Dresden, Germany; jens.goronzy@uniklinikum-dresden.de; 8 ARCUS Sports Clinic, 75179 Pforzheim, Germany; sobau@sportklinik.de; 9 Department of Orthopedic Surgery and Traumatology, St. Vinzenz-Hospital, 46535 Dinslaken, Germany; wolfgang@zinserweb.de

**Keywords:** femoroacetabular impingement, cartilage, patient-reported outcome, registry data

## Abstract

This study aims to report on a prospectively collected, multicenter database of patients undergoing hip arthroscopy for femoroacetabular impingement syndrome (FAI) and concomitant cartilage damage (according to the International Cartilage Repair Society) and to assess the outcome-affecting parameters. In the study, 353 hips with up to 24 months’ follow-up were assessed by iHOT-33 scoring and achievement of the minimal clinically important difference (MCID) and patient acceptable symptom state (PASS) levels. Multiple and binary regression analyses were performed to identify factors related to (un-) favorable outcomes and to assess their clinical relevance with regard to achieving the MCID and PASS. Multiple regression yielded the parameters of male sex (*p* = 0.022) and lower body mass index (BMI) (*p* = 0.019) at 6 months, lower BMI (*p* = 0.022) and younger age (*p* = 0.022) at 12 months, and younger age at 24 months (*p* = 0.039) to be significantly associated with higher iHOT scoring. Male sex (*p* = 0.019) and lower BMI (*p* = 0.018) were significantly correlated with achievement of the PASS in binary regression at 6 months, whereas at 12 (*p* = 0.010) and at 24 (*p* = 0.003) only younger age was shown to be significantly correlated. None of the parameters was statistically associated with achievement of the MCID. As the parameters of younger age, male sex, and lower BMI were identified as temporarily correlated with a preferable outcome in general and with achievement of the PASS in particular, these findings help to preoperatively identify factors associated with (un-) favorable therapy results.

## 1. Introduction

Femoroacetabular impingement syndrome (FAI) is a pre-arthritic condition caused by mechanically induced chondral defects in the hip [1,2,3,4], and its treatment by means of hip arthroscopy is routine nowadays [5,6,7,8,9].

While the overall favorable results of hip arthroscopy are largely undisputed, several parameters that affect the outcome of treatment have been identified. In the treatment of FAI, these include age, sex, and body mass index (BMI) [10]. Cvetanovich et al. reported on favorable outcomes in younger patients in terms of achieving the minimal clinically important difference (MCID) and patient acceptable symptom state (PASS) for the Hip Outcome Score (HOS) [11]. An age-dependent outcome was also reported by Gupta et al. and Nwachukwu et al. [12,13]. With regard to gender-related results, several authors reported more favorable outcomes for male patients. In fact, males outperformed age-adjusted females in several scores such as the HOS, modified Harris Hip Score (mHHS), and in terms of quality of life (QoL) [14,15,16,17]. A correlation between favorable therapy results and lower BMI, and, respectively, normal BMI has been repeatedly proven using these well-established scoring systems as well as a visual analog scale (VAS) [11,18,19].

In patients with FAI as their underlying diagnosis, concomitant intraarticular pathologies, especially chondral defects at the acetabulum, are a common finding [20,21]. This necessarily raises the question as to what extent concomitant cartilage damage may influence the outcome-affecting parameters already known for FAI. In this study, data from the German Cartilage Registry (KnorpelRegsister DGOU) were analyzed in order to present the treatment outcome and assess the outcome-affecting parameters, including their correlation to achievement of the MCID and the PASS in an FAI cohort with concomitant cartilage damage. It is hypothesized that the parameters of younger age, male sex, and lower BMI are correlated with a favorable outcome, whereas an increase in the cartilage defect-specific factors of lesion size and grade are associated with poorer treatment results.

## 2. Materials and Methods

This study is based on prospectively collected data from the German Cartilage Registry (KnorpelRegister DGOU), providing an evidence level of III. This database is a nationwide multicenter registry that was set up in 2013 to continuously follow up patients undergoing surgical cartilage therapy on the hip, knee, or ankle joint for multiple reasons. It is conducted in accordance with the Declaration of Helsinki and is listed in the German Clinical Trials Register (DRKS00005617). All patients included in the register signed written informed consent for participation. Pre and postoperative data collection was performed via a web-based remote data entry system. Baseline data as well as patient-reported outcome measure in the postoperative course were recorded. Perioperative data, such as defect- and joint-specific characteristics, as well as procedure-related information, were provided by the treating surgeon. The follow-up intervals used for this analysis were 6, 12, and 24 months after surgery. Participants were automatically contacted via email and asked to complete the questionnaires.

**Figure 1:**
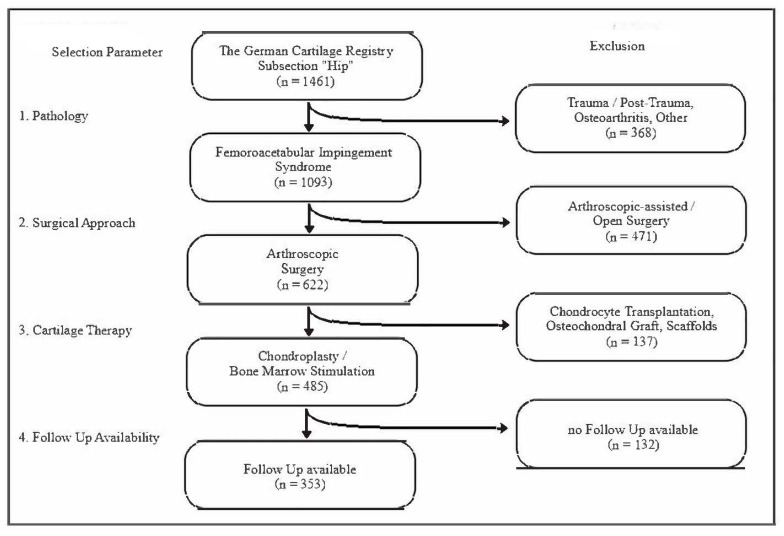
Patient selection procedure. Flowchart of the selection procedure based on the subsection ‘Hip’ of the German Cartilage Register (database as of August 2019). Note: “no Follow Up available” indicates an incomplete dataset.

By August 2019, the subsection “Hip” of the German Cartilage Registry included 1461 hips treated by surgery. For this study, subjects were selected according to their underlying pathology, the surgical approach, the type of cartilage therapy, and the availability of follow-up data. Only patients with FAI and accompanying chondral lesions that had been treated by hip arthroscopy were considered. Diagnoses were made by obligatory means of physical examination, conventional radiodiagnostics, and MRI. In order to form a homogenous study group and to reduce the influence of different types of cartilage therapy, only patients who had undergone ‘traditional’ treatment by means of chondroplasty (defined as debridement, contouring, or removal of devitalized/instable cartilage) or chondroplasty plus bone marrow stimulation (BMS; microfracturing/drilling) were included. In total, 353 hips were identified as eligible for inclusion in the current analysis. Details of the selection procedure are provided in Figure 1.

For the analysis of treatment outcome, the validated patient-reported outcome measure (PROM) “International Hip Outcome Tool” (iHOT-33) was used as it has been proven to be highly responsive to clinical change and particularly suitable for younger patients [22,23,24]. The iHOT-33 measure was calculated as the mean of the specific item responses ranging from 0 to 100, with 100 representing the best possible quality-of-life score. SPSS^®^ Statistics (Version 21.0.0.0, IBM^®^) was used for statistical analyses. Descriptive data are presented as mean (±standard deviation (SD)), percentage of the total, or in total numbers. Preoperative and postoperative iHOT-33 were compared by their differences in total scores using Student’s *t*-test for two dependent samples to check for significance. Postoperative scores were also assessed with regard to achievement of the iHOT’s MCID of 10 points and the PASS of 58 [23,25]. Cases of revision surgery in the postoperative course were recorded as well. Multiple regression analysis with backward stepwise elimination was then conducted to identify factors possibly affecting the postoperative iHOT-33 at 6, 12, and 24 months during the follow-up period. The independent variables therefore included age, sex, BMI, cartilage defect size, and cartilage defect stage (each as of time of surgery). With regard to the cartilage status, defect staging was carried out intraoperatively using the International Cartilage Repair Society classification system (ICRS) [26,27]. Subsequently, variables with significant results in the multiple regression were tested via binary logistic regression with backward elimination to assess if they were statistically related to successfully achieving the iHOT’s MCID and PASS. The preoperative iHOT was included in the analysis to check for possible tendencies towards achieving or failing the MCID and PASS in dependency on its baseline scores. *p*-values are two-sided and subject to a significance level of 0.05.

## 3. Results

### 3.1. Study Collective and Patient Demographics

The selection criteria identified 353 hips with a complete preoperative dataset and available follow-up data that were included in the study. The initial surgical intervention was performed between 2013 and 2019. Statistically significant differences between chondroplasty and chondroplasty + BMS with regard to age, BMI, and defect size were initially excluded. The mean age at the date of surgery was 38.6 ± 11.4 years. Of the 353 hips, 235 (66.6%) were male, 118 (33.4%) were female. Detailed information on the study cohort’s demographic and defect-specific baseline data is provided in Table 1. Based on the preoperative study collective, the following numbers of hips were available for follow-up: 303 (85.8%) at 6 months, 245 (69.4%) at 12 months, and 158 (44.8%) at 24 months. 

### 3.2. Functional Outcomes

**Figure 2:**
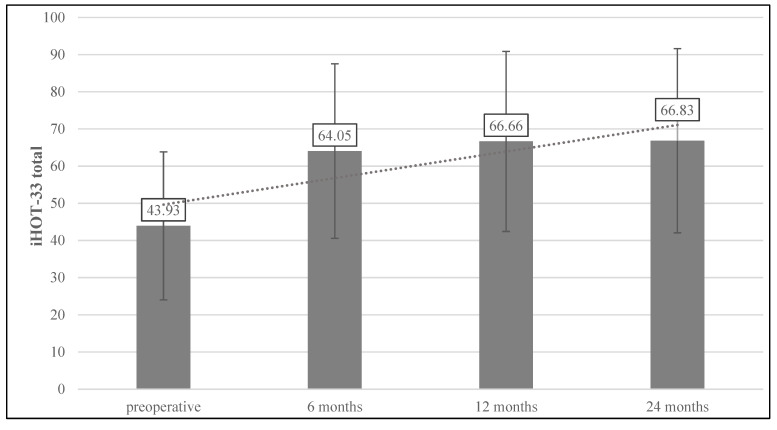
Pre- and postoperative course of the iHOT-33. Significant improvements in the mean outcome in the postoperative course compared to the mean preoperative score with each *p* < 0.001. iHOT-33, International Hip Outcome Tool (33 items).

**Figure 3:**
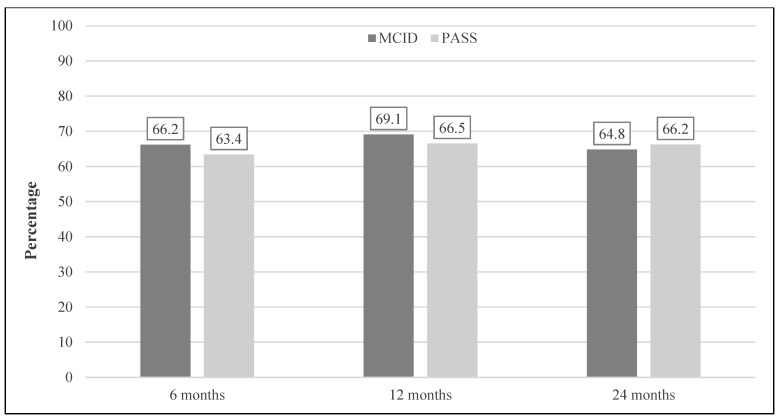
Achievement of the iHOT-33’s MCID and PASS. Postoperative course of hips achieving the iHOT-33’s MCID and PASS. iHOT-33, International Hip Outcome Tool (33 items); MCID, minimal clinically important difference; PASS, patient acceptable symptom state.

The preoperative mean iHOT-33 total score was 43.9 ± 19.9 for the whole study group, with 121 (34.3%) exceeding the PASS value. Postoperatively, the iHOT increased significantly from 64.1 ± 23.5 at 6 months to 66.8 ± 24.8 at 24 months (each *p* < 0.001) (Figure 2). The postoperative MCID was achieved by 66.2% of the group at the 6-month follow-up point and was stable at 64.8% at 24 months. The PASS score was exceeded by 63.4% at 6 months, with a trend towards further improvement during the follow-up period and 66.2% at 24 months (Figure 3).

### 3.3. Revision Surgery

During the follow-up period, 19 patients underwent further surgery on the initially treated hip, a total percentage of 5.4 for the whole study collective. Eight hips (2.3%) were converted to a total hip replacement, which was the most frequently performed reoperation. Details on the frequency and type of reoperations are provided in Table 2.

### 3.4. Multiple Regression Analysis of Factors Potentially Affecting the Postoperative iHOT-33

**Figure 4:**
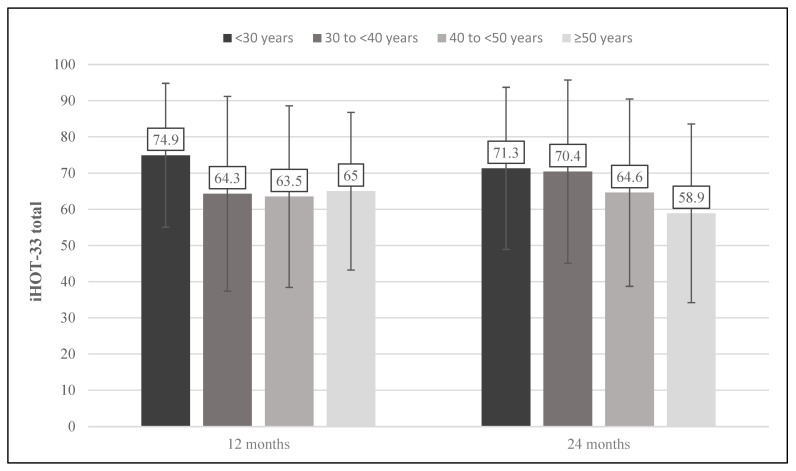
Age-dependent outcome. Age-dependent intra-follow-up differences in mean iHOT-33 scores at 12 and 24 months. iHOT-33, International Hip Outcome Tool (33 items).

Via multiple regression several parameters were identified as associated with a favorable treatment outcome. After 6 months’ follow-up, higher iHOT scores correlated with male sex (*p* = 0.022) and lower BMI (*p* = 0.019). After 12 months’ follow-up, the BMI remained significantly associated with better iHOT scores (*p* = 0.022) whereas gender-related results became statistically insignificant. Simultaneously, the analysis revealed a significant association between younger patients and increased postoperative iHOT scores between 12 (*p* = 0.022) and 24 months’ follow-up (*p* = 0.039). Neither the size nor the grade of cartilage defect was identified as significantly associated with higher or lower iHOT scores at any time during the follow-up period. Detailed data of the multiple regression analysis are provided in Table 3. Age-dependent intra-follow-up differences are displayed in Figure 4.

### 3.5. Binary Logistic Regression Analysis of Factors and Achievement of the MCID and the PASS

As the parameters of younger age, male sex, and lower BMI were revealed as at least temporarily associated with higher postoperative iHOT scores during the follow-up period, clinical relevance was assessed in terms of whether the iHOT’s MCID or PASS were achieved. Binary logistic regression showed that none of the abovementioned parameters were statistically associated with achievement of the MCID during the postoperative course. As concerns the PASS, male sex and lower BMI were found to be significantly associated with achievement of the PASS threshold after 6 months’ follow-up (*p*_sex_ = 0.019; *p*_BMI_ = 0.018). In the further follow-up course, the sole parameter of younger age was shown to be significantly associated with achievement of the iHOT’s PASS at 12 and 24 months postoperatively (*p*_12months_ = 0.010; *p*_24months_ = 0.003) (Table 4).

As far as the role of the preoperative iHOT-33 is concerned, logistic regression showed a significant association between lower values and achievement of the MCID at 6 months (*p* < 0.001), 12 months (*p* = 0.018), and 24 months (*p* = 0.008), respectively. Vice versa, higher preoperative iHOT scores were significantly and continuously correlated with achievement of the PASS threshold postoperatively (*p*_6months_ < 0.001; *p*_12months_ < 0.001; *p*_24months_ < 0.001).

## 4. Discussion

The assessment of outcome of hip arthroscopy for femoroacetabular impingement syndrome and its affecting parameters has recently been the focus of multiple publications [6,8,10,11,12,13,14,15,17,21]. This study provides an analysis of variables possibly affecting treatment outcome in patients with FAI and concomitant cartilage damage. In this context, the parameters of younger age, male sex, and lower BMI were found to be at least temporarily associated with favorable results during the follow-up course. These parameters also showed significance regarding the achievement of the iHOT’s PASS, whereas the proof of correlation with the achievement of the MCID failed. Finally, this study shows that factors possibly correlated with favorable/unfavorable outcomes do not necessarily remain constant, and may well change in the course of follow-up.

When comparing this study’s results with those reported by Griffin et al. in 2018, the mean improvements in iHOT scoring at 12 months’ follow-up appear to be similar, with a mean increase of 22.7 vs. 19.6 points. However, Griffin’s pre- and postoperative iHOT scores were generally lower than ours, with a 7.9 point difference at 12 months [9]. On the contrary, with a mean iHOT of 67 points at 12 months’ follow-up, our results were lower than those determined by Nwachukwu et al., who reported mean scores of 74 and 84, respectively [13,28]. This may be due to the mandatory cartilage defect in our study group in contrast to the comparative studies assessing the results of FAI treatment by hip arthroscopy in general. It is, however, also possible that those differences result from the database used for evaluation. Whereas Griffin et al. used a randomized controlled trial design, Nwachukwu calculated results from an institutional registry [9,13,28]. Therefore, highly selected patients on the one hand and data from a single high-volume center on the other hand might have affected the results obtained [29].

With regard to the iHOT’s MCID of 10 points and PASS value of 58, the 66.2% (MCID) and 63.4% (PASS) achieved by our study group already met these criteria at first follow-up. These results showed a stable value or a trend toward subsequent improvement in the further course [23,25]. Similar findings have been reported, showing that more than half of the patients treated with hip arthroscopy for FAI achieve the MCID at six months’ follow-up [30]. However, our MCID percentages were again lower than those reported by Nwachukwu et al. during the follow-up periods of six months (66 vs. 76) and 12 months (69 vs. 85), which again might be related to the concomitant cartilage damage but to patient selection as well [28,29]. Comparable studies using other PROM, such as HOS or mHHS, also report a tendency for higher percentages of patients achieving the MCID, and the results concerning the PASS appear to be similar [11,12,31]. The overall percentage of reoperations in this study was 5.9%, which fairly accurately matches previously published results by Cvetanovich et al. and Migliorini and Maffulli, who reported overall rates of revision surgery of 5.6% and 5%, respectively [8,11]. Thus, it appears that initial concomitant cartilage damage does not increase the frequency of reoperations postoperatively.

The current study’s findings on outcome-affecting parameters showed a statistically significant correlation of favorable results with younger age, male sex, and lower BMI. These factors had previously been identified in reviews by Levy et al. and Sogbein et al., but also in more specific research papers [10,11,12,13,14,15,18,19]. However, this study provides the results of an FAI cohort with proven cartilage damage, which undoubtedly has the potential to influence outcome-affecting parameters. Moreover, previously identified factors had usually been calculated at a single endpoint. The current study provides results that not only identify outcome-affecting parameters throughout a follow-up period from 6 to 24 months, but also documents how they changed during this period. Multiple regression, for instance, showed that male sex was only correlated with increased iHOT scoring and achievement of the PASS value at six months’ follow-up. In contrast to this, Malviya et al. reported on male patients who scored significantly better than females in terms of quality of life at 12 months postoperatively [15]. Frank et al. reported that females scored worse in HOS and mHHS at 24 months follow-up, but only those aged 45 years or more [14]. The question remains as to how far sex can independently affect therapy results. In their recent comprehensive analysis on gender-based differences of FAI therapy outcome with a mean follow-up of 4.3 years, Maerz et al. principally confirmed that males perform better in postoperative PROM. However, they also proved that this was primarily due to lower scoring in preoperative PROM in females and not connected to sex as an individual parameter, which supports the findings of Joseph et al. already published in 2016 [17,32].

As regards the role of BMI in treatment outcome, multiple regression yielded a significant correlation of lower BMI and higher iHOT scores at 6 and 12 months postoperatively. In terms of clinical significance, logistic regression only proved that lower BMI was correlated with achievement of the PASS at 6 months’ follow-up, whereas correlation to achievement of the MCID could not be shown. Levy et al., on the contrary, reported lower BMI, which was associated with achievement of the MCID in the HOS score (Activities of Daily Living & Sports-Specific Subscale) after a minimum follow-up of one year [19]. Cvetanovich et al. also identified lower BMI as associated with favorable outcome in terms of achievement of the MCID, although only in the sports-specific HOS and at two years’ follow-up [11]. Neither publication reported on the effects regarding the achievement of the PASS [11,19]. These differences may be due to the type of patients selected for our study group, again emphasizing the significance of concomitant cartilage damage, but as well to the different PROM chosen to evaluate treatment outcome. 

As far as the parameter of age is concerned, the correlation between younger age and positive treatment results has repeatedly been reported [11,12,13]. From the opposite perspective, inferior outcomes have been published, particularly in patients aged ≥50 years [12,33,34]. Therefore, our findings generally confirm those of other studies. However, this study also shows that the parameter of age is not immediately linked to treatment outcome, but becomes relevant from the 12-month follow-up point onward. Beyond that, clinical significance is gained by the calculation of a statistical correlation between younger age and the achievement of the PASS score at 12 and 24 months postoperatively. In their crucial paper defining the substantial clinical benefit (SCB) in FAI treatment, Nwachukwu et al. reported that younger age was an independent predictor for positive treatment results in patients with intact cartilage status [13]. This is particularly important as the iHOT’s SCB of 63.5 points in Nwachukwu’s study only slightly exceeds its PASS of 58 points, whereas the mean scores in our study continuously exceed the SCB and PASS throughout the whole follow-up period. Moreover, Nwachukwu et al. were able to prove the parameter age to be independent of the preoperative functional status [13]. In summary, by proving a significant correlation between younger age and the achievement of the PASS, this study delivers results similar to those of Nwachukwu. So, despite concomitant cartilage damage, the likelihood of achieving a satisfactory postoperative symptom state is greater in younger patients. However, the MCID was not proven to be statistically correlated with the parameter age. This is in contrast to the findings of Cvetanovich et al., who identified a significant correlation between younger age and achievement of the MCID at 24 months’ follow-up (HOS; Activities of Daily Living and Sports-Specific Subscale) [11]. Again, it only can be assumed that besides patient selection, concomitant cartilage damage in FAI patients might alter statistical correlations concerning achievement of the iHOT’s MCID. Nevertheless, it was Nwachukwu et al. who showed that FAI patients without chondral defects perform better in postoperative functional scoring [30].

Interestingly, and refuting the initial hypothesis for the defect-specific parameters, neither the extent nor the grade of concomitant cartilage damage statistically affected the postoperative iHOT scores and, consequently, whether the MCID or PASS were achieved or not. Moreover, although not the primary focus of this study, the relevance of preoperative iHOT scores must not be neglected. In fact, lower preoperative scores are significantly to highly significantly associated with achievement of the MCID in the postoperative course, whereas higher preoperative scores are similarly correlated to achieving the PASS. This has been previously reported and therefore appears to apply for FAI patients with concomitant cartilage defects as well [10,11,12,15,17,21].

There are limitations to this study that must have to be taken into account when interpreting the presented results. First and most important, this study’s results are based on registry data. This provides the possibility to assess a large, multicenter database, which may reflect a ‘real-world’ practice setting more accurately compared to randomized controlled trials [29]. However, registry data generally are characterized by greater patient heterogeneity and strongly depend on the parameters recorded/not recorded. For this study, there is a selection bias as only patients who had undergone primary hip arthroscopy to treat isolated FAI and concomitant cartilage damage were included. This was deliberate, in order to form a homogenous study group, but the results might have been different for other inclusion criteria. The same applies for the type for cartilage treatment, where results could have been different depending on the chosen type of therapy. There is also a reduction in the number of patients available in the course of follow-up, which is due to the ongoing process of following-up patients in the German Cartilage Registry. However, absolute patient counts in the respective follow-up generally allow for the statistical calculations that were performed. With regard to parameters that were not routinely recorded, it is worth mentioning the possible influence of concomitant labral treatment as the different types are known to affect the overall results of FAI therapy [35,36]. In addition, the presence of osteoarthritis might alter clinical results as well, as radiographic degeneration is a predictor of poorer outcomes [37,38,39]. Finally, baseline parameters such as BMI may have changed during the follow-up course. Possible effects on the results cannot be excluded with complete certainty.

## 5. Conclusions

Patients undergoing hip arthroscopy for FAI and concomitant cartilage damage experienced significant and stable improvement in functional outcome during the postoperative course from 6 to 24 months when using iHOT-33 as the primary outcome measure. The percentages of patients achieving the MCID and PASS tend to be lower than in comparable studies not including cartilage damage. During the entire follow-up course, the parameters of younger age, male sex, and lower BMI were identified as at least temporarily correlated with a favorable outcome in general and achievement of the PASS in particular. This study’s findings help to preoperatively identify factors associated with (un-)favorable treatment results.

## Figures and Tables

**Table 1:** Baseline demographics. Baseline demographics and defect-specific characteristics (ICRS, International Cartilage Repair Society).

Parameter		Mean ± SD or n (%)
Age, years Sex, male/female Body Mass Index, kg/m^2^ Cartilage defect size, mm^2^ Cartilage defect grade, ICRS	I II III IV	38.6 ± 11.4 235 (66.6)/118 (33.4) 25.9 ± 9.1 162.5 ± 169.9 25 (7.1) 83 (23.5) 187 (53.0) 58 (16.4)

**Table 2:** Revision surgery. Frequency and type of revision surgery on the initially treated hip during the follow-up period.

Procedure	6 Months	12 Months	24 Months	Total
	n (% of Follow up group)			n (% of Study group)
Revision hip arthroscopy Total hip arthroplasty Others	2 (0.7) 2 (0.7) 2 (0.7) (1× Lipoma, 1× Osteonecrosis)	2 (0.8) 1 (0.4) 2 (0.8 1× Scar revision, 1× n.a.)	2 (1.3) 5 (3.2) 1 (0.6) (1× Derotation osteotomy)	6 (1.7) 8 (2.3) 5 (1.4)
Total	6 (2.0)	5 (2.0)	8 (5.1)	19 (5.4)

**Table 3:** Analysis of correlation with postoperative iHOT-33. Multiple regression analysis (backward stepwise elimination) of patient- and defect-specific factors with potential influence on the postoperative iHOT-33 total. *b*, Regression coefficient; SE (*b*), Standard error of *b*; *p*, *p*-value; ICRS, International Cartilage Repair Society; R^2^, Coefficient of determination; iHOT-33, International Hip Outcome Tool (33 items); Note: * *p* ≤ 0.05, ** *p* ≤ 0.01, *** *p* ≤ 0.001.

Parameter	iHOT-33 Total		
	6 Months	12 Months	24 Months
Age *b* (default) *b* (robust) SE(*b*) *p* Sex (coding: 1 male, 2 female) *b* (default) *b* (robust) SE(*b*) *p* Body mass index *b* (default) *b* (robust) SE(*b*) *p* Cartilage defect size *b* (default) *b* (robust) SE(*b*) *p* Cartilage defect grade, ICRS *b* (default) *b* (robust) SE(*b*) *p* Constant *b* (default)	Eliminated from model −0.665 * −0.139 * 0.289 0.022 −0.087 * −0.142 * 0.037 0.019 Eliminated from model Eliminated from model 9.577 ***	−0.033 * −0.151 * 0.014 0.022 −0.596 −0.151 0.331 0.073 −0.033 * −0.151 * 0.014 0.022 Eliminated from model Eliminated from model 9.638 ***	−0.037 * −0.173 * 0.018 0.039 Eliminated from model Eliminated from model Eliminated from model Eliminated from model 8.161 ***
R^2^ Adjusted R^2^ F Statistic	0.030 0.023 4.400 * (df = 2; 281)	0.062 0.049 4.897 ** (df = 3; 223)	0.030 0.023 4.358 * (df = 1; 141)

**Table 4:** Analysis of correlation with postoperative MCID and PASS. Binary logistic regression analysis (backward stepwise elimination) of factors identified as significantly affecting postoperative iHOT-33 and achievement of the MCID and the PASS. iHOT-33, International Hip Outcome Tool (33 items); MCID, minimal clinically important difference; PASS, patient acceptable symptom state.

Parameter		Odds Ratio	95% CI	*p*-Value
MCID 6 months PASS 6 months MCID 12 months PASS 12 months MCID 24 months PASS 24 months	Sex Body Mass Index Sex Body Mass Index Age Body Mass Index Age Body Mass Index Age Age	1.24 0.98 0.54 0.93 1.00 0.97 0.97 0.98 0.98 0.95	0.71–2.16 0.91–1.05 0.32–0.91 0.87–0.99 0.98–1.03 0.92–1.03 0.94–0.99 0.94–1.01 0.95–1.02 0.92–0.98	0.453 0.499 0.019 * 0.018 * 0.751 0.316 0.010 ** 0.209 0.304 0.003 **

Note: Odds ratio for age and body mass index are per unit change in continuous variables; * *p* ≤ 0.05, ** *p* ≤ 0.01.

## Data Availability

The data that supports the findings of this study are available from the corresponding author upon reasonable request.

## References

[1] Leunig M., Beaulé P.E., Ganz R. (2009). The Concept of Femoroacetabular Impingement: Current Status and Future Perspectives. Clin. Orthop. Relat. Res..

[2] Beck M., Kalhor M., Leunig M., Ganz R. (2005). Hip morphology influences the pattern of damage to the acetabular cartilage. J. Bone Jt. Surg..

[3] Agricola R., Heijboer M.P., Bierma-Zeinstra S.M.A., Verhaar J.A.N., Weinans H., Waarsing J.H. (2013). Cam impingement causes osteoarthritis of the hip: A nationwide prospective cohort study (CHECK). Ann. Rheum. Dis..

[4] Clohisy J.C., Dobson M.A., Robison J.F., Warth L.C., Zheng J., Liu S.S., Yehyawi T.M., Callaghan J.J. (2011). Radiographic Structural Abnormalities Associated with Premature, Natural Hip-Joint Failure. J. Bone Jt. Surg..

[5] Fayad T.E., Khan M.A.A., Haddad F.S. (2013). Femoroacetabular impingement. Bone Jt. J..

[6] Minkara A.A., Westermann R., Rosneck J., Lynch T.S. (2019). Systematic Review and Meta-analysis of Outcomes after Hip Arthroscopy in Femoroacetabular Impingement. Am. J. Sports Med..

[7] Claßen T., Körsmeier K., Kamminga M., Beck S., Rekowski J., Jäger M., Landgraeber S. (2016). Is early treatment of cam-type femoroacetabular impingement the key to avoiding associated full thickness isolated chondral defects?. Knee Surg. Sports Traumatol. Arthrosc..

[8] Migliorini F., Maffulli N. (2021). Arthroscopic Management of Femoroacetabular Impingement in Adolescents: A Systematic Review. Am. J. Sports Med..

[9] Griffin D.R., Dickenson E.J., Wall P., Achana F., Donovan J.L., Griffin J., Hobson R., Hutchinson C.E., Jepson M., Parsons N. (2018). Hip arthroscopy versus best conservative care for the treatment of femoroacetabular impingement syndrome (UK FASHIoN): A multicentre randomised controlled trial. Lancet.

[10] Sogbein O.A., Shah A., Kay J., Memon M., Simunovic N., Belzile E.L., Ayeni O.R. (2019). Predictors of Outcomes After Hip Arthroscopic Surgery for Femoroacetabular Impingement: A Systematic Review. Orthop. J. Sports Med..

[11] Cvetanovich G.L., Weber A.E., Kuhns B., Alter J., Harris J.D., Mather I.R.C., Nho S.J. (2017). Hip Arthroscopic Surgery for Femoroacetabular Impingement with Capsular Management: Factors Associated with Achieving Clinically Significant Outcomes. Am. J. Sports Med..

[12] Gupta A., Redmond J.M., Stake C.E., Dunne K.F., Domb B.G. (2015). Does Primary Hip Arthroscopy Result in Improved Clinical Outcomes? 2-Year Clinical Follow-up on a Mixed Group of 738 Consecutive Primary Hip Arthroscopies Performed at a High-Volume Referral Center. Am. J. Sports Med..

[13] Nwachukwu B.U., Chang B., Fields K., Rebolledo B.J., Nawabi D.H., Kelly B.T., Ranawat A.S. (2017). Defining the “Substantial Clinical Benefit” After Arthroscopic Treatment of Femoroacetabular Impingement. Am. J. Sports Med..

[14] Frank R.M., Lee S., Bush-Joseph C.A., Salata M.J., Mather R.C., Nho S.J. (2016). Outcomes for Hip Arthroscopy According to Sex and Age. A Comparative Matched-Group Analysis. J. Bone Jt. Surg..

[15] Malviya A., Stafford G.H., Villar R.N. (2012). Impact of arthroscopy of the hip for femoroacetabular impingement on quality of life at a mean follow-up of 3.2 years. J. Bone Jt. Surg. Br. Vol..

[16] Philippon M.J., Ejnisman L., Ellis H.B., Briggs K.K. (2012). Outcomes 2 to 5 Years Following Hip Arthroscopy for Femoroacetabular Impingement in the Patient Aged 11 to 16 Years. Arthrosc. J. Arthrosc. Relat. Surg..

[17] Maerz T., Nepple J.J., Bedi A., Zaltz I., Belzile E., Beaulé P.E., Sink E.L., Clohisy J.C. (2021). Sex Differences in Clinical Outcomes Following Surgical Treatment of Femoroacetabular Impingement. J. Bone Jt. Surg..

[18] Saltzman B.M., Kuhns B.D., Basques B., Leroux T., Alter J., Mather R.C., Salata M.J., Nho S.J. (2017). The Influence of Body Mass Index on Outcomes After Hip Arthroscopic Surgery with Capsular Plication for the Treatment of Femoroacetabular Impingement. Am. J. Sports Med..

[19] Levy D.M., Kuhns B., Chahal J., Philippon M.J., Kelly B.T., Nho S.J. (2016). Hip Arthroscopy Outcomes with Respect to Patient Acceptable Symptomatic State and Minimal Clinically Important Difference. Arthrosc. J. Arthrosc. Relat. Surg..

[20] Lynch T.S., Terry M.A., Bedi A., Kelly B.T. (2013). Hip Arthroscopic Surgery. Am. J. Sports Med..

[21] Philippon M.J., Briggs K., Yen Y.-M., Kuppersmith D.A. (2009). Outcomes following hip arthroscopy for femoroacetabular impingement with associated chondrolabral dysfunction. J. Bone Jt. Surg..

[22] Mohtadi N.G., Griffin D.R., Pedersen M.E., Chan D., Safran M.R., Parsons N., Sekiya J.K., Kelly B.T., Werle J.R., Leunig M. (2012). The Development and Validation of a Self-Administered Quality-of-Life Outcome Measure for Young, Active Patients with Symptomatic Hip Disease: The International Hip Outcome Tool (iHOT-33). Arthrosc. J. Arthrosc. Relat. Surg..

[23] Baumann F., Weber J., Zeman F., Muller M., Lahner M., Nerlich M., Fickert S. (2015). Validation of a German version of the International Hip Outcome Tool (G-iHOT33) according to the COSMIN checklist: How much improvement is clinically relevant?. Arch. Orthop. Trauma. Surg..

[24] Dwyer M.K., Green M., McCarthy J.C. (2015). Assessing outcomes following arthroscopic labral debridement--what can the IHOT-33 reveal?. J. Hip Preserv. Surg..

[25] Maxwell S., Pergaminelis N., Renouf J., Tirosh O., Tran P. (2018). Identification of a Patient Acceptable Symptomatic State Score for the International Hip Outcome Tool in People Undergoing Hip Arthroscopy. Arthrosc. J. Arthrosc. Relat. Surg..

[26] Smith G.D., Taylor J., Almqvist K.F., Erggelet C., Knutsen G., Portabella M.G., Smith T., Richardson J.B. (2005). Arthroscopic Assessment of Cartilage Repair: A Validation Study of 2 Scoring Systems. Arthrosc. J. Arthrosc. Relat. Surg..

[27] Brittberg M., Winalski C.S. (2003). Evaluation of Cartilage Injuries and Repair. J. Bone Jt. Surg..

[28] Nwachukwu B.U., Chang B., Kahlenberg C.A., Fields K., Nawabi D.H., Kelly B.T., Ranawat A.S. (2017). Arthroscopic Treatment of Femoroacetabular Impingement in Adolescents Provides Clinically Significant Outcome Improvement. Arthrosc. J. Arthrosc. Relat. Surg..

[29] De Geus S.W., Sachs T.E., Tseng J.F. (2020). Big Data vs. Clinical Trials in HPB Surgery. J. Gastrointest. Surg..

[30] Nwachukwu B.U., Chang B., Adjei J., Schairer W.W., Ranawat A.S., Kelly B.T., Nawabi D.H. (2018). Time Required to Achieve Minimal Clinically Important Difference and Substantial Clinical Benefit After Arthroscopic Treatment of Femoroacetabular Impingement. Am. J. Sports Med..

[31] Chahal J., van Thiel G.S., Mather I.R.C., Lee S., Song S.H., Davis A., Salata M., Nho S.J. (2015). The Patient Acceptable Symptomatic State for the Modified Harris Hip Score and Hip Outcome Score Among Patients Undergoing Surgical Treatment for Femoroacetabular Impingement. Am. J. Sports Med..

[32] Joseph R., Pan X., Cenkus K., Brown-Taylor L., Ellis T., Di Stasi S. (2015). Sex Differences in Self-Reported Hip Function Up to 2 Years After Arthroscopic Surgery for Femoroacetabular Impingement. Am. J. Sports Med..

[33] Bryan A.J., Krych A.J., Reardon P.J., Berardelli R., Pareek A., Levy B.A. (2016). Are Short-term Outcomes of Hip Arthroscopy in Patients 55 Years and Older Inferior to Those in Younger Patients?. Am. J. Sports Med..

[34] Domb B.G., Linder D., Finley Z., Botser I.B., Chen A., Williamson J., Gupta A. (2015). Outcomes of Hip Arthroscopy in Patients Aged 50 Years or Older Compared with a Matched-Pair Control of Patients Aged 30 Years or Younger. Arthrosc. J. Arthrosc. Relat. Surg..

[35] Ortiz-Declet V., Mu B., Chen A.W., Litrenta J., Perets I., Yuen L.C., Domb B.G. (2018). Should the Capsule Be Repaired or Plicated After Hip Arthroscopy for Labral Tears Associated with Femoroacetabular Impingement or Instability? A Systematic Review. Arthrosc. J. Arthrosc. Relat. Surg..

[36] Larson C.M., Giveans M.R., Stone R.M. (2012). Arthroscopic Debridement Versus Refixation of the Acetabular Labrum Associated with Femoroacetabular Impingement. Am. J. Sports Med..

[37] Nakashima H., Utsunomiya H., Kanezaki S., Suzuki H., Nakamura E., Larson C.M., Sakai A., Uchida S. (2021). Is Arthroscopic Hip Labral Repair/Reconstruction Surgery Effective for Treating Femoroacetabular Impingement in the Presence of Osteoarthritis?. Clin. J. Sport Med..

[38] Larson C.M., Giveans R.M., Taylor M. (2011). Does Arthroscopic FAI Correction Improve Function with Radiographic Arthritis?. Clin. Orthop. Relat. Res..

[39] Horisberger M., Brunner A., Herzog R.F. (2010). Arthroscopic Treatment of Femoral Acetabular Impingement in Patients with Preoperative Generalized Degenerative Changes. Arthrosc. J. Arthrosc. Relat. Surg..

